# Supplementary material for: Inhibition of Biofilm Formation by Modified Oxylipins from the Shipworm Symbiont Teredinibacter turnerae
Source: Mar Drugs. 2020 Dec 20;18(12):656. doi: 10.3390/md18120656 (PMC7766104; doi:10.3390/md18120656)
Supplement: Supplementary file 1 [file marinedrugs-18-00656-s001.pdf]

Supplemental Data

Title: Inhibition of Biofilm Formation by Modified Oxylipins from the Shipworm Symbiont *Teredinibacter turnerae*

Authors: Noel M. Lacerna II[1], Cydeee Marie V. Ramones[1], Jose Miguel D. Robes[1], Jortan O. Tun[1], Myra Ruth A. Picart[1], Bailey Miller[2], Margo G. Haygood[2], Eric W. Schmidt[2], Lilibeth A. Salvador-Reyes[1] and Gisela P. Concepcion[1]

Affiliations:[1]The Marine Science Institute, University of the Philippines Diliman, Quezon City, Philippines 1101, [2]Department of Medicinal Chemistry, University of Utah, Salt Lake City, Utah, USA

Contents:

Figure S1. HRMS spectrum of turneroic acid (**1**) $[M+Na]^+$ in positive mode.

Figure S2. ^1H NMR Spectrum of turneroic acid (**1**) in CD_3OD (500 MHz).

Figure S3. COSY Spectrum of turneroic acid (**1**) in CD_3OD (500 MHz).

Figure S4. HSQC Spectrum of turneroic acid (**1**) in CD_3OD (500 MHz).

Figure S5. HMBC Spectrum of turneroic acid (**1**) in CD_3OD (500 MHz).

Figure S6. TOCSY Spectrum of turneroic acid (**1**) in CD_3OD (500 MHz).

Figure S7. LC- ESIMS of Compound **2** $[M+H]^+$ = 269.131, $C_{16}H_{28}O_3$

Figure S8. LC- ESI-MS of Compound **3** $[M+H]^+$ = 297.171, $C_{18}H_{32}O_3$

Figure S9. MS/MS of **2** at 20V in positive mode.

Figure S10. MS/MS of **3** at 20V in positive mode.

Figure S11. ^1H NMR spectrum of **2** in CD_3OD (500 MHz).

Figure S12. ^1H NMR spectrum of **3** in CD_3OD (500 MHz).

Figure S13. Concentration dependent curve of **1** and Dispersin B against *S. epidermidis* biofilm formation and planktonic cells.

Figure S14. Concentration dependent curve of **2** and **3** against *S. epidermidis* biofilm formation and planktonic cells.

Figure S15. Concentration dependent curve of **4**, **5**, **6**, and **7** against *S. epidermidis* biofilm formation and planktonic cells.

Figure S16. Concentration dependent curve of oxacillin against *S. aureus* and MRSA.

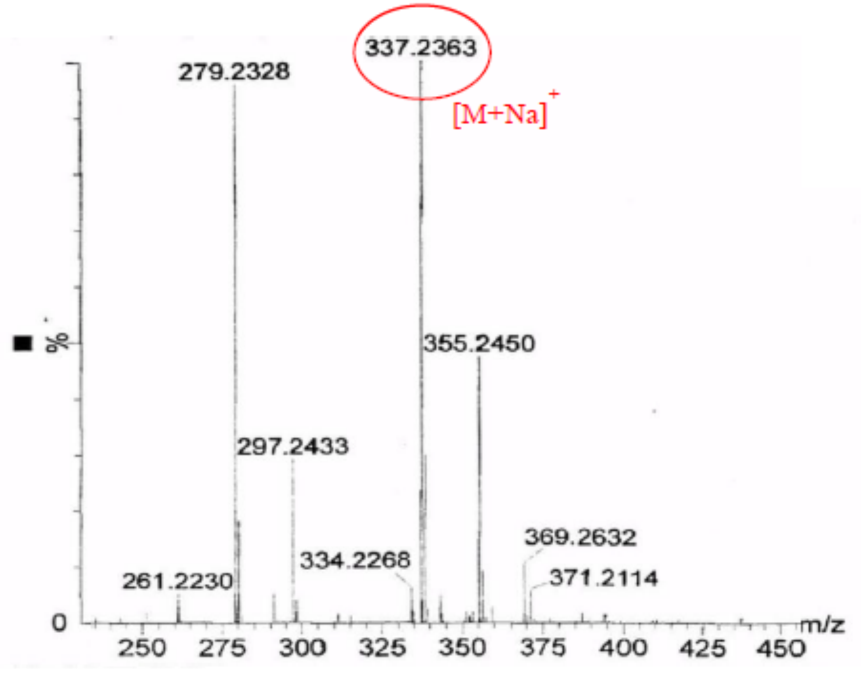

Figure S1. HRMS spectrum of turneroic acid (**1)** [M+Na]+ in positive mode.

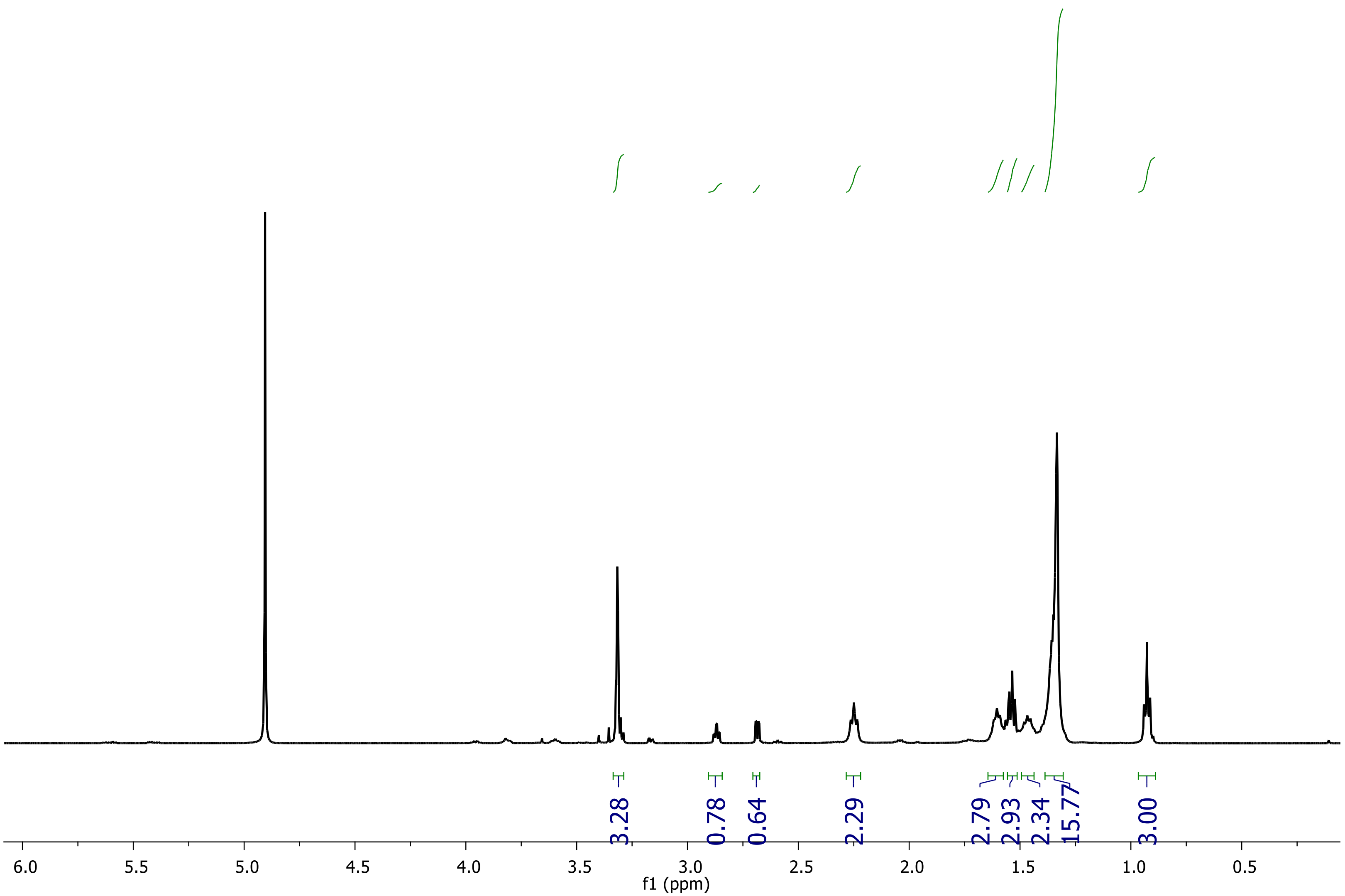

Figure S2. ^{1}H NMR Spectrum of turneroic acid (**1**) in CD_3OD (500 MHz).

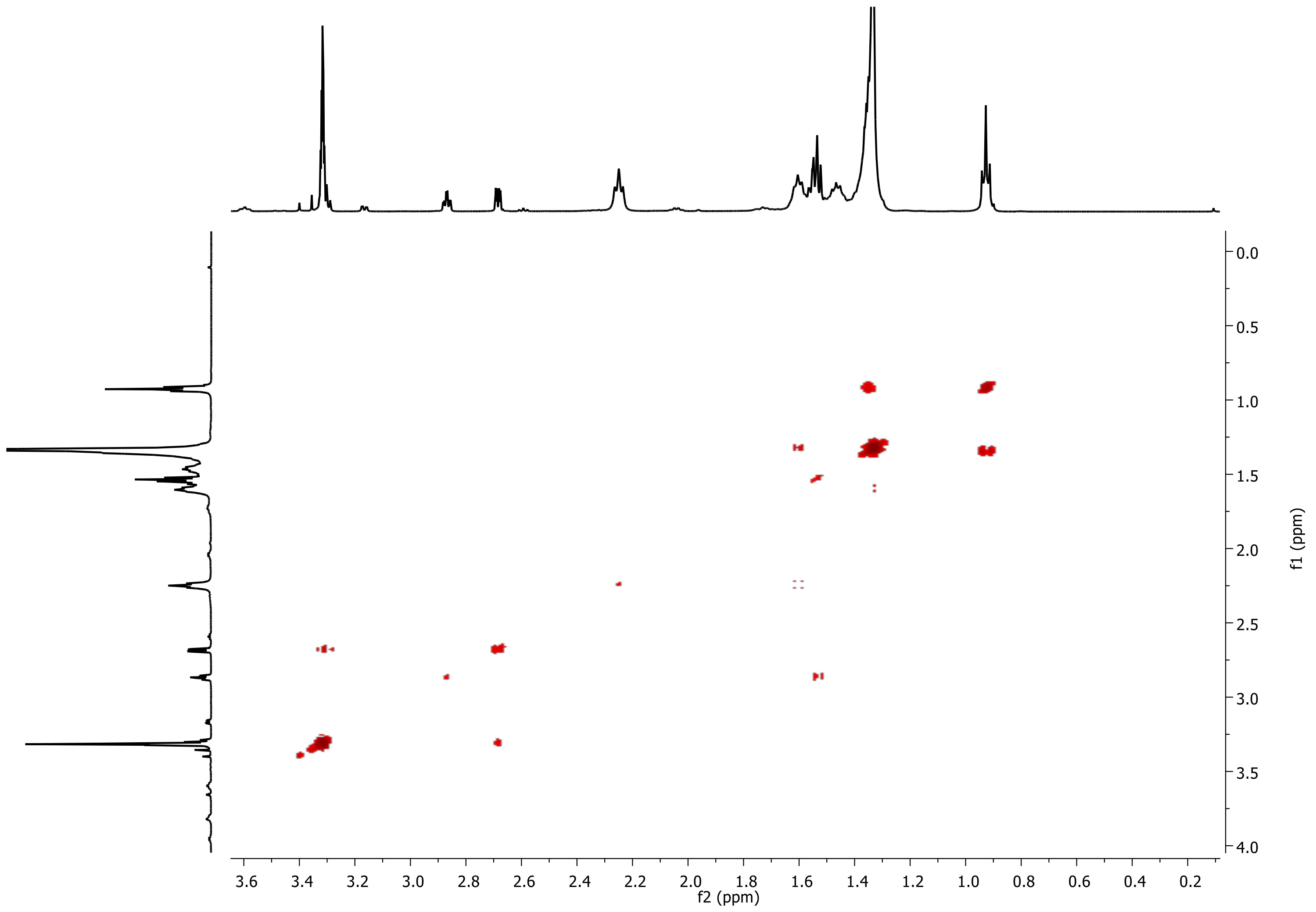

Figure S3. COSY Spectrum of turneroic acid (**1**) in CD_3OD (500 MHz).

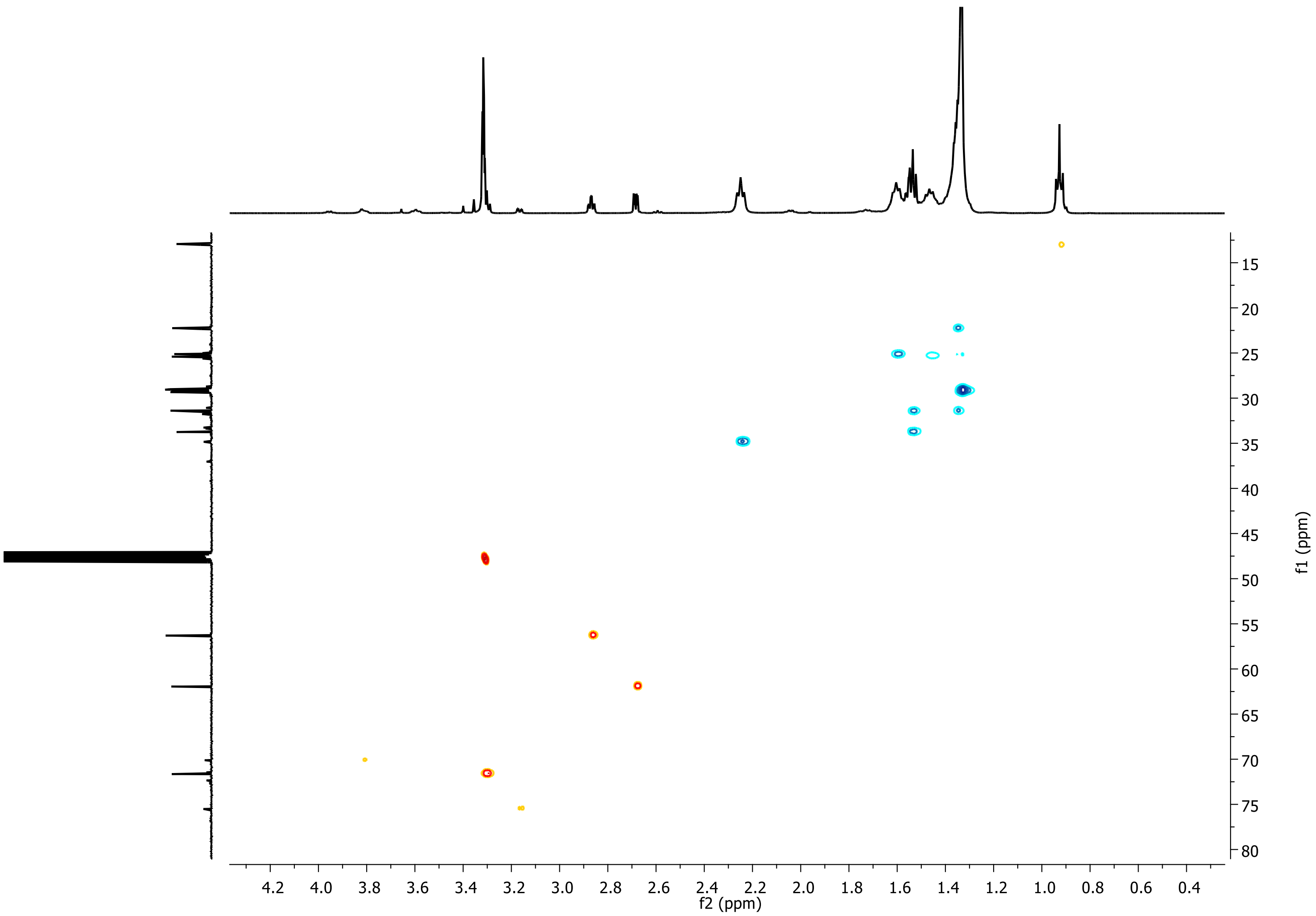

Figure S4. HSQC Spectrum of turneroic acid (**1**) in CD_3OD (500 MHz).

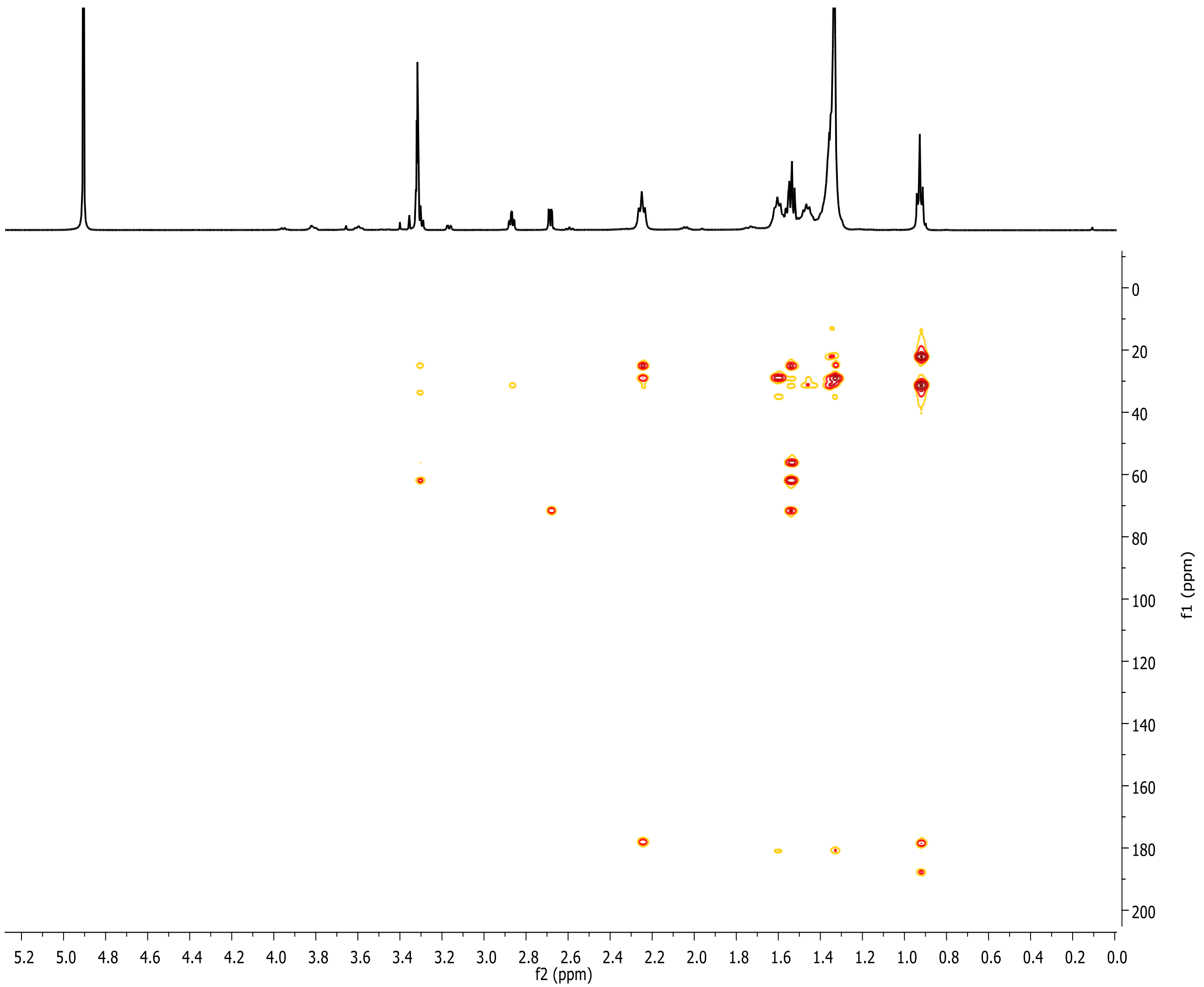

Figure S5. HMBC Spectrum of turneroic acid (**1**) in CD_3OD (500 MHz).

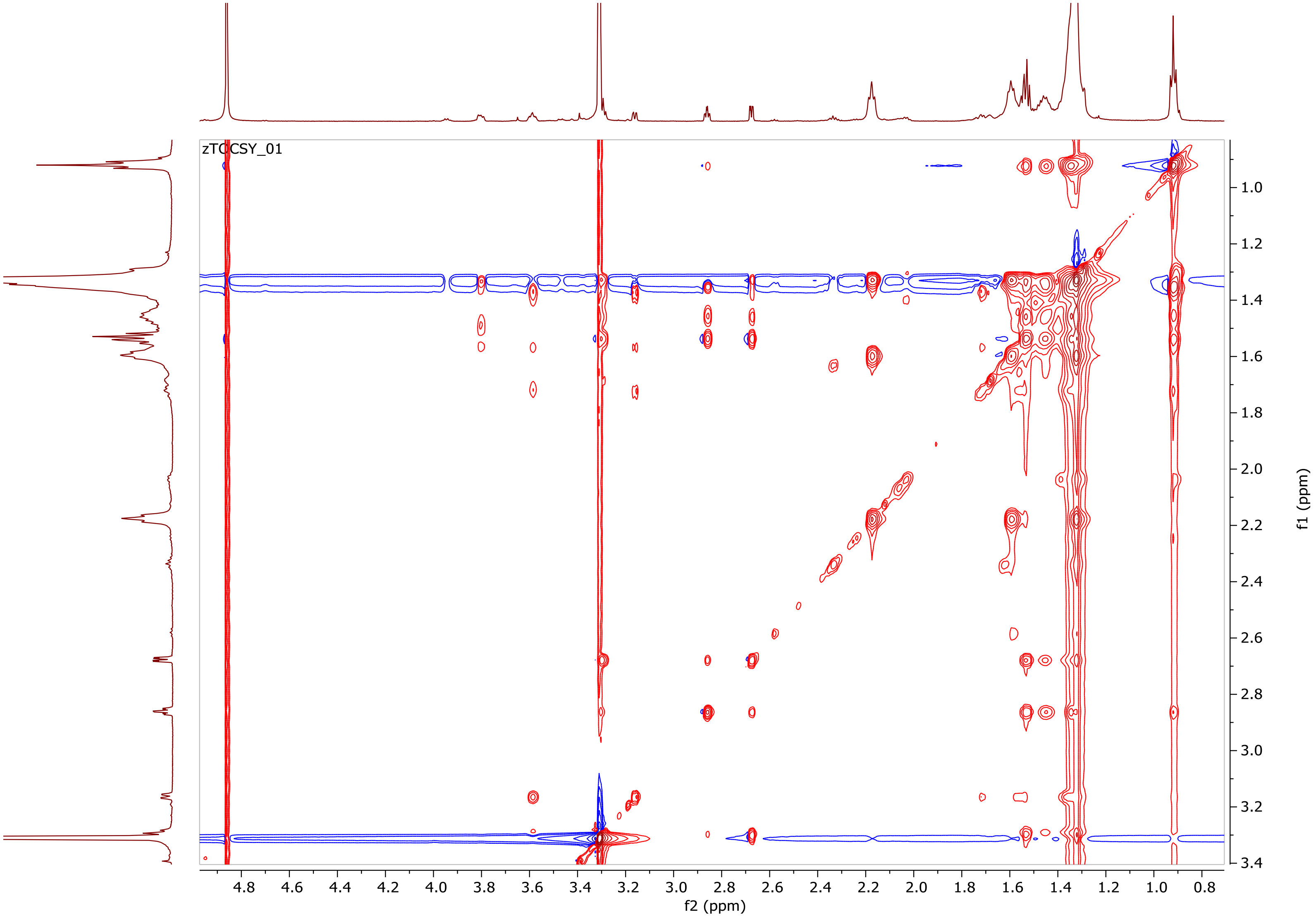

Figure S6. TOCSY Spectrum of turneroic acid (**1**) in CD_3OD (500 MHz). The TOCSY spectrum showed the presence of minor components in turneroic acid after prolonged storage in solution.

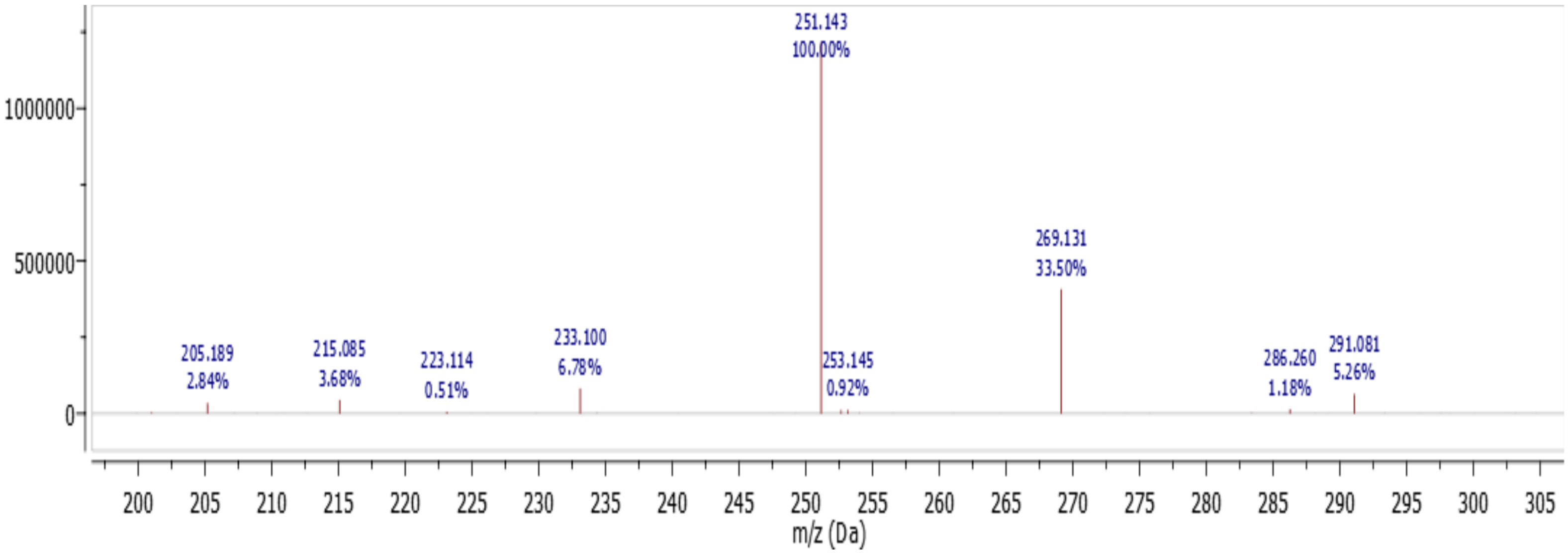

Figure S7. LC- ESIMS of **2** $[M+H]^+$ = 269.131, $C_{16}H_{28}O_3$

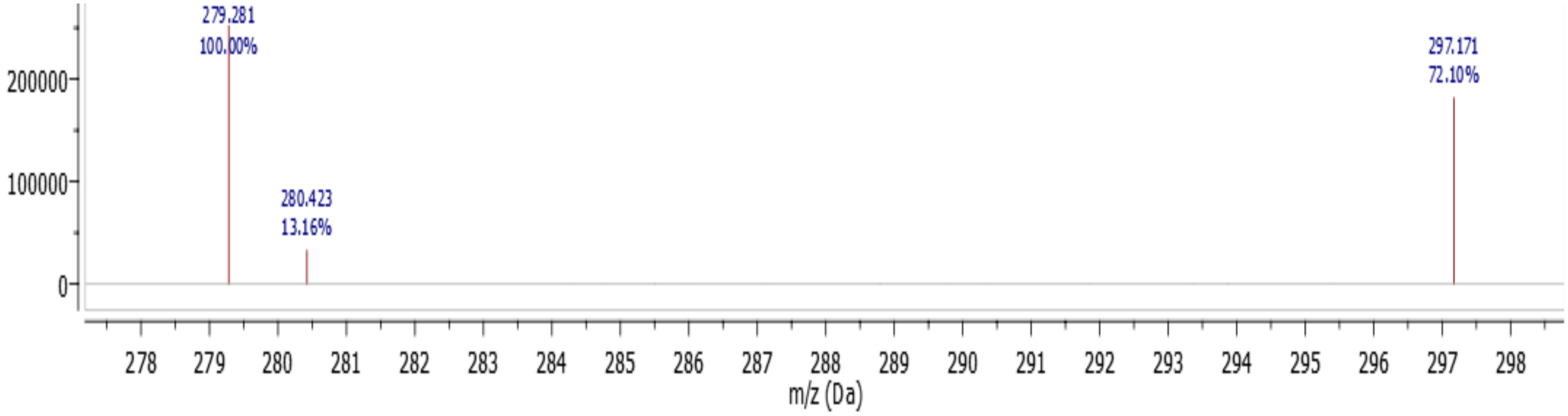

Figure S8. LC- ESIMS of **3** $[M+H]^+$ = 297.171, $C_{18}H_{32}O_3$

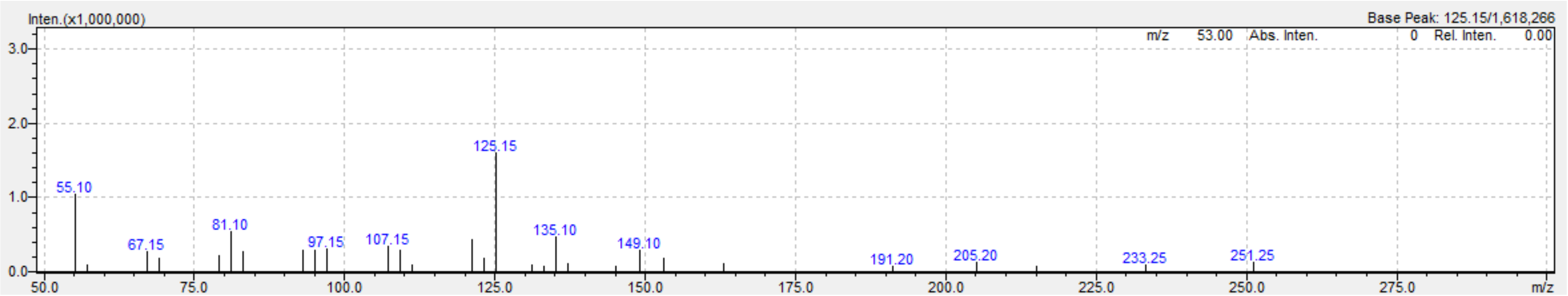

Figure S9. MS/MS of **2** at 20V in positive mode.

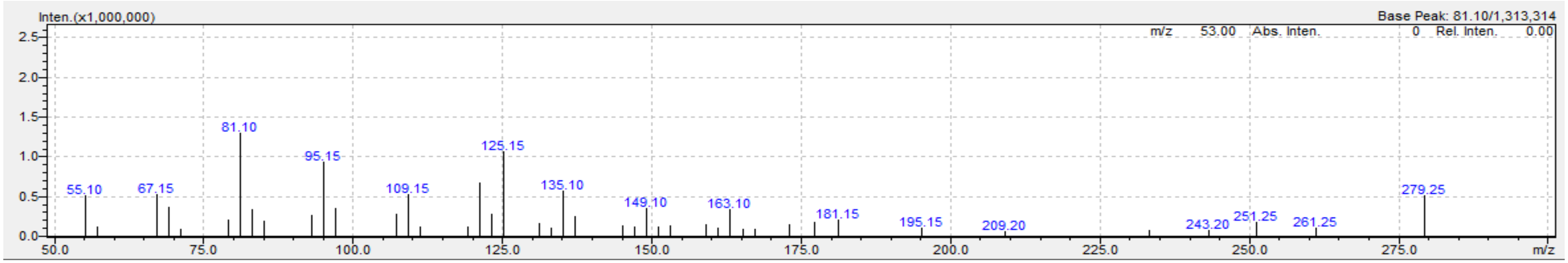

Figure S10. MS/MS of **3** at 20V in positive mode.

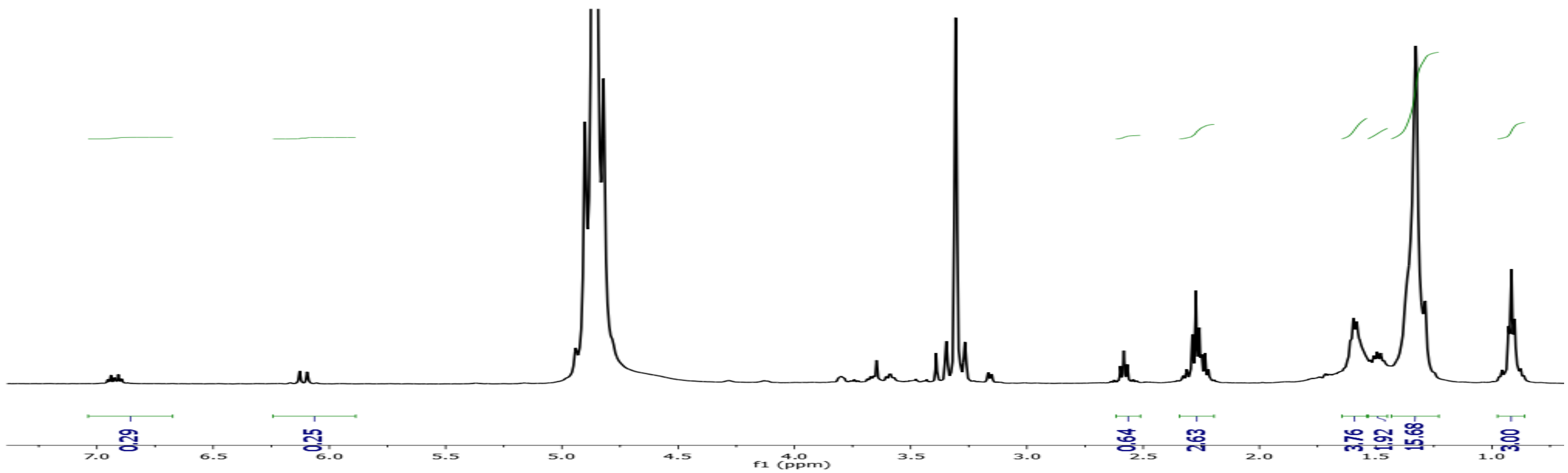

Figure S11. ^{1}H NMR spectrum of **2** in CD_3OD (500 MHz).

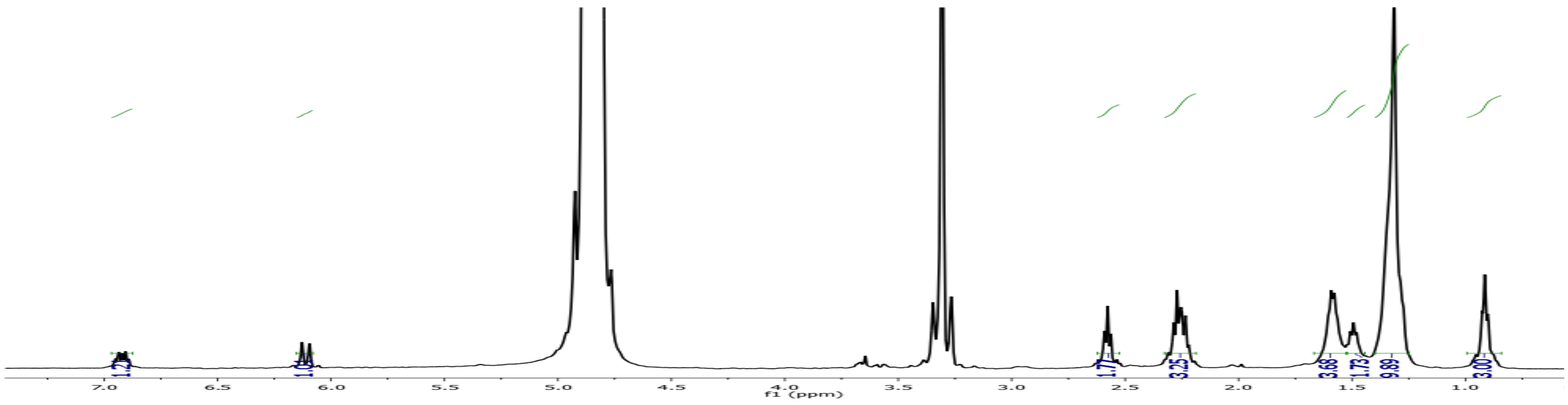

Figure S12. ^{1}H NMR spectrum of **3** in CD_3OD (500 MHz).

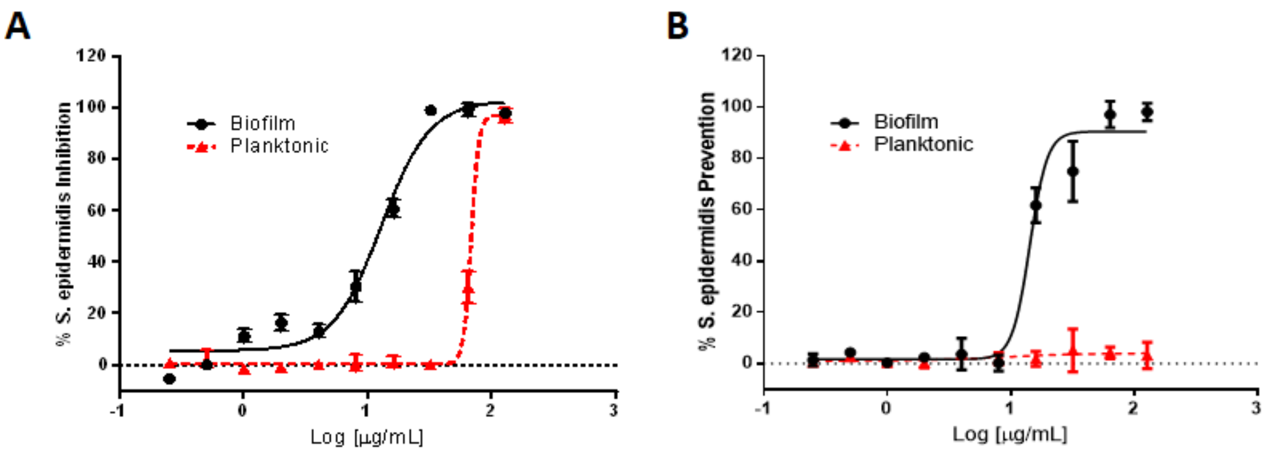

Figure S13. Concentration dependent response curve of **1** (A) and Dispersin B (B) tested against *S. epidermidis* RP62A (ATCC 35984) biofilm formation and planktonic cells. Compounds were tested in a two-fold dilution scheme from 0.25 – 128 µg/mL. Data presented as mean % inhibition ± SD of two independent trials with four independent replicates.

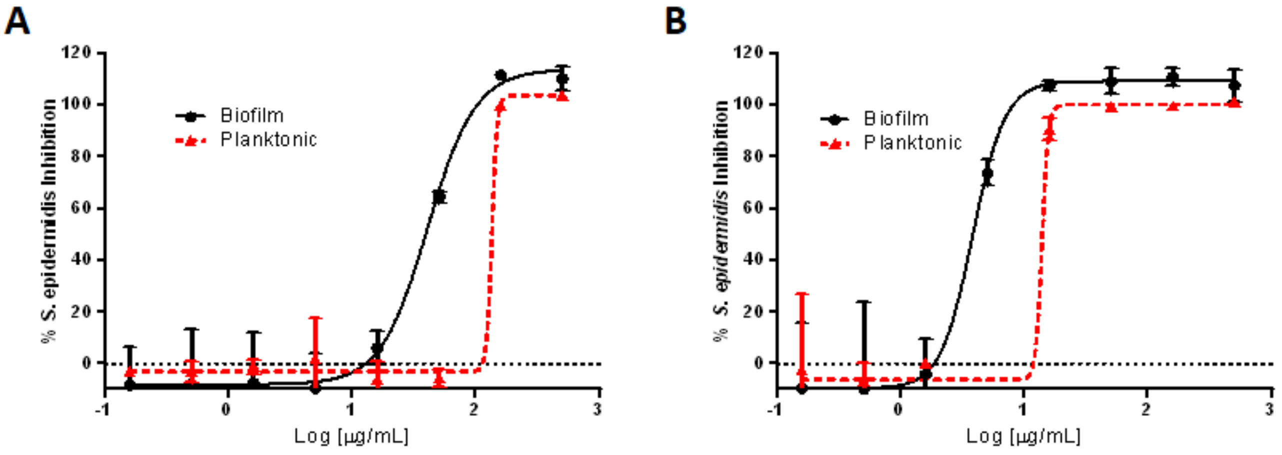

Figure S14. Concentration dependent response curve of **2** (A) and **3** (B) tested against *S. epidermidis* RP62A (ATCC 35984) biofilm formation and planktonic cells. Compounds were tested in a half-log dilution scheme from 0.158 – 500 µg/mL. Data presented as mean % inhibition ± SD of two independent trials with four independent replicates.

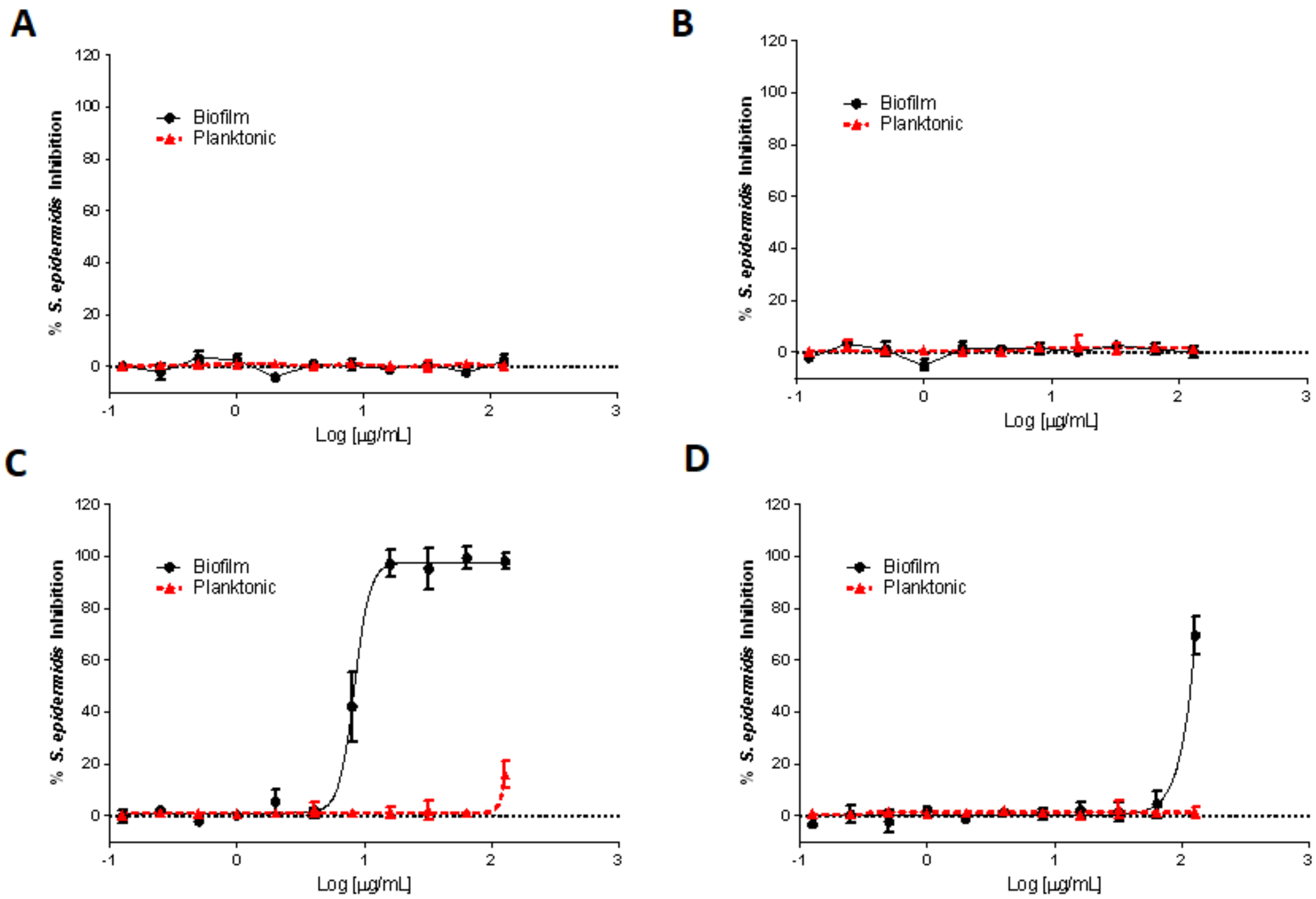

Figure S15. Concentration dependent response curve of **4** (A), **5** (B), **6** (C), and **7** (D) tested against *S. epidermidis* RP62A (ATCC 35984) biofilm formation and planktonic cells. Compounds were tested in a two-fold dilution scheme from 0.25 – 128 µg/mL. Data presented as mean % inhibition ± SD of two independent trials with four independent replicates.

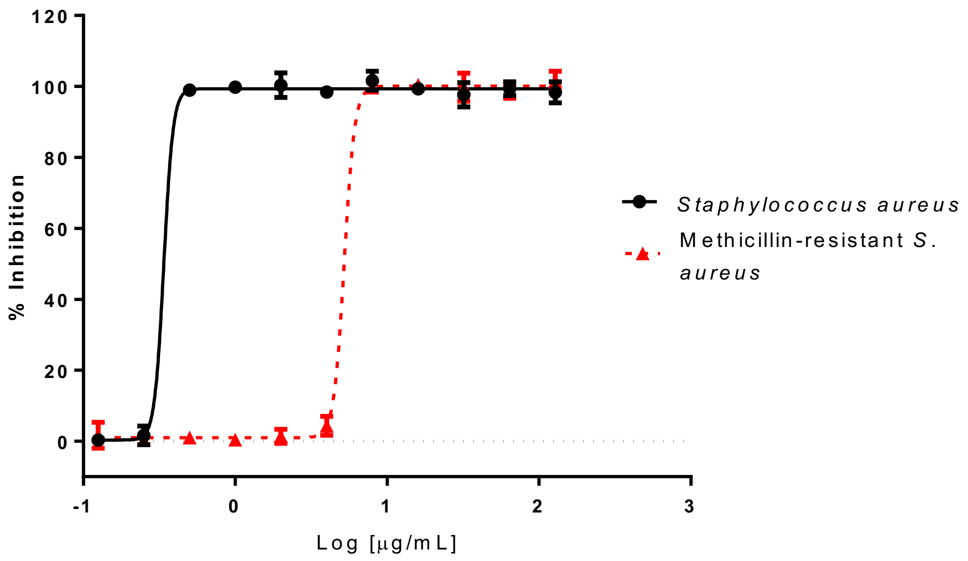

Figure S16. Concentration dependent response curve of oxacillin tested against *S. aureus* (ATCC6538) and methicillin-resistant *S. aureus* (ATCC43300). Compounds were tested in a two-fold dilution scheme from 0.125 – 128 µg/mL. Data presented as mean % inhibition ± SD of two independent trials with four independent replicates.
